# Frailty trajectories and associated factors in the years prior to death: evidence from 14 countries in the Survey of Health, Aging and Retirement in Europe

**DOI:** 10.1186/s12877-023-03736-1

**Published:** 2023-01-27

**Authors:** Natalie D. Jenkins, Miles Welstead, Lucy Stirland, Emiel O. Hoogendijk, Joshua J. Armstrong, Annie Robitaille, Graciela Muniz-Terrera

**Affiliations:** 1 Edinburgh Dementia Prevention, University of Edinburgh, Edinburgh, Scotland; 2 Glasgow Brain Injury Research Group, University of Glasgow, Glasgow, Scotland; 3 Department of Epidemiology & Biostatistics, Amsterdam Public Health Research Institute, Amsterdam UMC – Location VU University Medical Center, Amsterdam, the Netherlands; 4 Department of Health Sciences, Lakehead University, Thunder Bay, ON Canada; 5 Faculty of Health Sciences, University of Ottawa, Ottawa, Canada; 6 Department of Neurology, Oregon Health & Science University, Portland, OR USA; 7 Department Social Medicine, Ohio University, Athens, USA

**Keywords:** Frailty trajectories, Longitudinal data, Mortality, End of life care, Age-related changes

## Abstract

**Background:**

Age-related changes in frailty have been documented in the literature. However, the evidence regarding changes in frailty prior to death is scarce. Understanding patterns of frailty progression as individuals approach death could inform care and potentially lead to interventions to improve individual’s well-being at the end of life. In this paper, we estimate the progression of frailty in the years prior to death.

**Methods:**

Using data from 8,317 deceased participants of the Survey of Health, Ageing, and Retirement in Europe, we derived a 56-item Frailty Index. In a coordinated analysis of repeated measures of the frailty index in 14 countries, we fitted growth curve models to estimate trajectories of frailty as a function of distance to death controlling both the level and rate of frailty progression for age, sex, years to death and dementia diagnosis.

**Results:**

Across all countries, frailty before death progressed linearly. In 12 of the 14 countries included in our analyses, women had higher levels of frailty close to the time of death, although they progressed at a slower rate than men (e.g. Switzerland (-0.008, SE = 0.003) and Spain (-0.004, SE = 0.002)). Older age at the time of death and incident dementia were associated with higher levels and increased rate of change in frailty, whilst higher education was associated with lower levels of frailty in the year preceding death (e.g. Denmark (0.000, SE = 0.001)).

**Conclusion:**

The progression of frailty before death was linear. Our results suggest that interventions aimed at slowing frailty progression may need to be different for men and women. Further longitudinal research on individual patterns and changes of frailty is warranted to support the development of personalized care pathways at the end of life.

**Supplementary Information:**

The online version contains supplementary material available at 10.1186/s12877-023-03736-1.

## Introduction

Frailty is a state of increased vulnerability to adverse outcomes in older populations resulting from disorders of several physiological systems [1]. A growing body of literature is emerging regarding aging-related frailty progression, and on the identification of factors that may modify these trajectories [2–4]. A recent systematic review by Welstead et al., identified several significant risk and protective factors for frailty progression [5]. For example, an important body of literature documents gender differences in frailty, demonstrating that women typically have higher levels of frailty than men [3, 6, 7]. Furthermore, evidence suggests that older women have higher levels of disability, comorbidities and polypharmacy than men [8]. Yet, somewhat paradoxically, women have longer life expectancies than men. This suggests that women live for a longer period of time with higher levels of frailty, a well-documented discrepancy between health and survival between men and women, termed the ‘male-female health survival paradox’ [8, 9]. Amongst other factors, frailty progression has also been found to be associated with brain pathologies, particularly Alzheimer’s Disease [10, 11], older age [4, 12] and geographical location [3, 13]. For instance, in European countries, Stolz et al. show that frailty trajectories were steeper for those living in Southern European countries than countries further north [3]. Despite a growing body of literature looking into frailty progression, there is still a distinct lack of research examining frailty trajectories and modifying factors towards the end of life.

In a recent review of end of life trajectories, Cohen-Mansfield et al. [14] reported that the majority of findings regarding terminal changes focus on cognitive or functional decline, although changes in other aspects of health and wellbeing have also been studied. Further, they identified a gap in knowledge regarding the progression of frailty at the end of life. Although some studies have examined frailty cross-sectionally as a predictor of mortality [15], studies of frailty trajectories in the years prior to death are lacking. Stolz et al. [16] show an accelerated increase in the frailty index (FI) during the last years of life occurring approximately three years prior to death, with decline beginning earlier in women than men. A second study examined changes in FI in four population-based cohorts and demonstrated that individuals with a steeper increase in FI had a higher mortality risk in all four cohorts [17]. The next step in this line of research is to begin exploring the factors that influence these end-of-life frailty trajectories. Gaining a better understanding of frailty progression at the end of life and the factors that may slow its progression will help to inform care at the end of life and improve quality of life [18–21].

In this study, we examine trajectories of frailty in the years prior to death, in participants with and without dementia, from 14 countries participating in the Survey of Health, Aging and Retirement Study in Europe (SHARE). Our hypotheses are: (1) frailty increases linearly and not in a curvilinear manner as individuals approach death; (2) sociodemographic factors such as education and sex, as well as dementia, modify the level and rate of progression of frailty and (3) that there are differences across countries in frailty progression.

## Methods

### Data

SHARE is a pan-European longitudinal survey of health and economic factors in community dwelling individuals aged 50 years and over [22, 23], harmonised with the Health and Retirement Study (HRS) and the English Longitudinal Study of Ageing (ELSA), two large studies conducted in the US and England respectively. The database provides harmonised data from over 530,000 interviews conducted on approximately 140,000 participants from 27 European countries and Israel from 2004 to 2017. Study participants answer questions about health and health care, income and wealth, work and retirement and social networks at various study waves using face to face computer-assisted interviewing, combined with self-completion questionnaires. Mortality data, including year of death, is captured using end-of-life interviews completed by a proxy-respondent in the close social network of the deceased. Further details regarding data collection are described elsewhere [22, 23].

The present study used SHARE data from five waves over a period of 14 years to calculate a FI at each time point. Data from Wave 3 was not included due to lack of essential variables. Mortality data, specifically age at death, from Waves 2–6 (release version 6.1.1 19th June 2018), and Wave 7 (release version 7.0.0 3rd April 2019) were included.

In order to estimate non-linear trajectories of acceleration of change [24], we selected all countries from SHARE with at least three waves of FI data (Austria, Belgium, Czech Republic, Denmark, France, Germany, Greece, Israel, Italy, Poland, Slovenia, Spain, Sweden, Switzerland). The Netherlands was not included as FI data was available for fewer than 25 participants at more than two time points, which was considered insufficient for estimating trajectories of change. See Table 1 for descriptive characteristics.

**Table 1:** Descriptive characteristics of the analytic sample

	Sample (N)										
	Time Point 1	Time Point 2	Time Point 3	Time Point 4	Time Point 5	Age at Baseline (SD)	FI at Baseline (SD)	Years of Education (SD)	Male %	Dementia %	Years to Death at baseline (SD)
**Northern Europe**											
** Sweden**	716	412	177	90	29	76.1 (9.99)	0.22 (0.17)	9.14 (3.17)	47.10	10.10	5.56 (3.57)
** Denmark**	606	373	201	106	28	73.87 (10.29)	0.21 (0.15)	11.32 (3.69)	50.70	6.30	5.50 (3.59)
**Western Europe**											
** Austria**	544	331	147	46	20	73.51 (10.00)	0.24 (0.17)	8.72 (4.19)	52.30	12.70	5.54 (3.32)
** Belgium**	655	461	257	129	61	74.58 (10.79)	0.26 (0.17)	9.77 (4.24)	49.70	10.50	5.13 (3.37)
** France**	563	350	190	81	28	75.05 (10.88)	0.26 (0.16)	7.52 (4.64)	47.80	9.20	5.08 (3.44)
** Germany**	328	154	52	31	13	72.1 (10.80)	0.26 (0.18)	12.1 (3.55)	42.10	7.90	3.51 (3.19)
** Switzerland**	275	180	94	27	10	75.39 (10.27)	0.17 (0.13)	8.48 (4.65)	41.10	8.40	4.75 (3.06)
**Eastern Europe**											
** Czech Republic**	811	455	178	37	-	73.07 (10.04)	0.27 (0.17)	11.23 (3.04)	48.20	8.60	3.86 (2.56)
** Poland**	429	222	68	-	-	71.33 (9.90)	0.31 (0.18)	7.84 (3.08)	47.00	9.30	5.46 (2.69)
**Southern Europe**											
** Greece**	641	424	109	-	-	76.47 (8.54)	0.23 (0.16)	6.41 (4.10)	53.80	5.00	7.49 (3.59)
** Italy**	715	464	257	139	41	73.30 (9.34)	0.27 (0.18)	6.14 (4.00)	46.70	9.80	5.87 (3.64)
** Slovenia**	341	175	66	-	-	76.13 (10.18)	0.28 (0.17)	9.34 (3.51)	45.60	12.90	2.67 (1.90)
** Spain**	1191	741	357	172	47	76.71 (10.33)	0.31 (0.20)	6.08 (4.57)	47.00	18.40	4.96 (3.53)
** Israel**	502	293	133	45	-	74.60 (9.87)	0.32 (0.20)	9.68 (4.84)	42.40	12.70	5.66 (3.22)

*SD *Standard deviation

Participants were included in the analyses if they were: deceased with no missing data regarding year of death; and aged 50 and older and without dementia at study entry. At each wave, SHARE recruits a refreshment sample for the purposes of refreshing the longitudinal cohort and compensating for attrition, furthermore, on occasion follow up visits at subsequent waves are missed. These two factors result in various wave participation patterns between participants. We report the baseline visit as the first visit attended by the participant. Further time points indicate sequential follow up visits irrespective of wave number.

### Frailty index

The FI is a commonly used tool which conceptualises frailty as an accumulation of deficits across multiple body systems (e.g. physical, psychosocial, cognitive) [25]. The standard procedure for creating an FI is outlined by Searle et al. [26]. It is recommended that at least 30 deficits are chosen to optimise the validity of the measure. Previous research has found consistent reliability in the predictive value of multiple FIs even when different deficits are included in their composition [27]. The FI is calculated as an individual’s total number of deficits on a continuous scale from 0 to 1 with higher values indicating higher frailty [1]. Following this approach, symptoms, signs, diseases, and disabilities are considered as deficits in health which accumulate over time in relation to age. Using data from each wave, we constructed a 56-item FI by modifying the existing 70-item SHARE-FI created by Theou et al. [28]. This was chosen over existing SHARE-FI versions for two reasons. Firstly, to allow us to remove deficits related to cognitive health as this study planned to analyse the FI in relation to dementia status. Since cognitive decline is a key clinical feature of dementia, including cognitive FI deficits would confound the analyses. Secondly, to ensure that all items included were available at each wave for a consistent longitudinal FI, and in each country, thereby created a harmonised analytic approach which enables the direct comparison of FI trajectories between countries.

The 56-item FI included self-reported measures of mental health, functional abilities, physical health and signs and symptoms associated with ageing (See Table S1 for full detail).

### Other variables

Years to death was calculated as the difference between age at study entry and age of death in years. This allowed us to code distance to death using negative values to correspond with progression of time from left to right.

Other key explanatory variables included in our analyses were: sex/gender (male-female/men-women) [8, 9]; self-reported incidence of dementia [10, 29]; education, recorded as self-report years of full-time education; and age at baseline recorded in years. Data collection of sex and gender are not clearly delineated in SHARE, sex is recorded as interviewer observation where interviewers are prompted to ask if unsure. As such, the distinction between biological sex and gender is unfeasible, necessitating the use of the combined association. We include the term sex/gender to model the combined association of biological and social mechanisms, both of which may contribute to frailty trajectories in women. Sex/gender was recoded as 0 for male and 1 for female. Distance to death from study entry was also included in the models to understand differences between individuals who join the study closer to death and those who join the study further from death.

### Statistical analysis

For each country, we fit independent latent growth curve models to repeated measures of the FI structured as a function of distance to death in years. We estimated models describing both linear and quadratic trajectories of change, including random effects for the level and slope parameters to allow for heterogeneity across individuals. Level and slope parameters were adjusted for age at baseline (centered at age 65), education (centered at 7 years), distance to death from study entry (centered at 5 years), sex (coded as 1 = female, 0 = male), and an indicator variable of incident dementia (1 = incident case, 0 = no case). To test whether there was a nonlinear association of age at baseline with frailty level and slope, we also included a term for the squared value of age at baseline. The intercept of the models was set at 6 months before death. With these specifications, the intercept of the linear growth models represent the mean value of the FI, at half a year before death, for a reference individual (that is, for a man aged 65 years, with seven years of education, who entered the study five years prior to death, and did not receive a diagnosis of dementia during follow up). For the linear model, the linear slope represents the mean rate of increase in FI per year closer to death. In quadratic models, the linear slope represents the rate of change at half year before death for a reference individual and the quadratic term, the change in the rate of change in FI in the years before death. Due to lack of sufficient data on more than three timepoints, quadratic trajectories of change were not modelled in Poland, Greece and Slovenia.

**Fig. 1:**
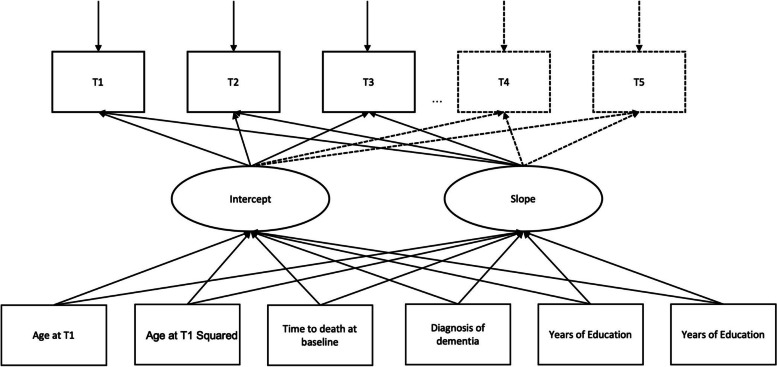
A path diagram representing the final models for each country. The dotted lines represent the additional timepoints in countries with longer follow-up periods. Each timepoint (T) is measured as the number of years to death at each follow-up visit

After fitting models that estimated linear and quadratic trajectories to each of the datasets, we compared Bayesian Information Criterion (BIC, [30]) indices from both models. Models were estimated using Mplus 8.1 [31, 32]. The final models are represented in Fig. 1, where observed data is depicted as rectangles, and latent variables are depicted as ovals. The top row of rectangles represents the time metric used in the model (time to death at each data collection wave) whereas the bottom row of rectangles represent the variables used to adjust the intercept and slope parameters to control for between person differences.

**Fig. 2:**
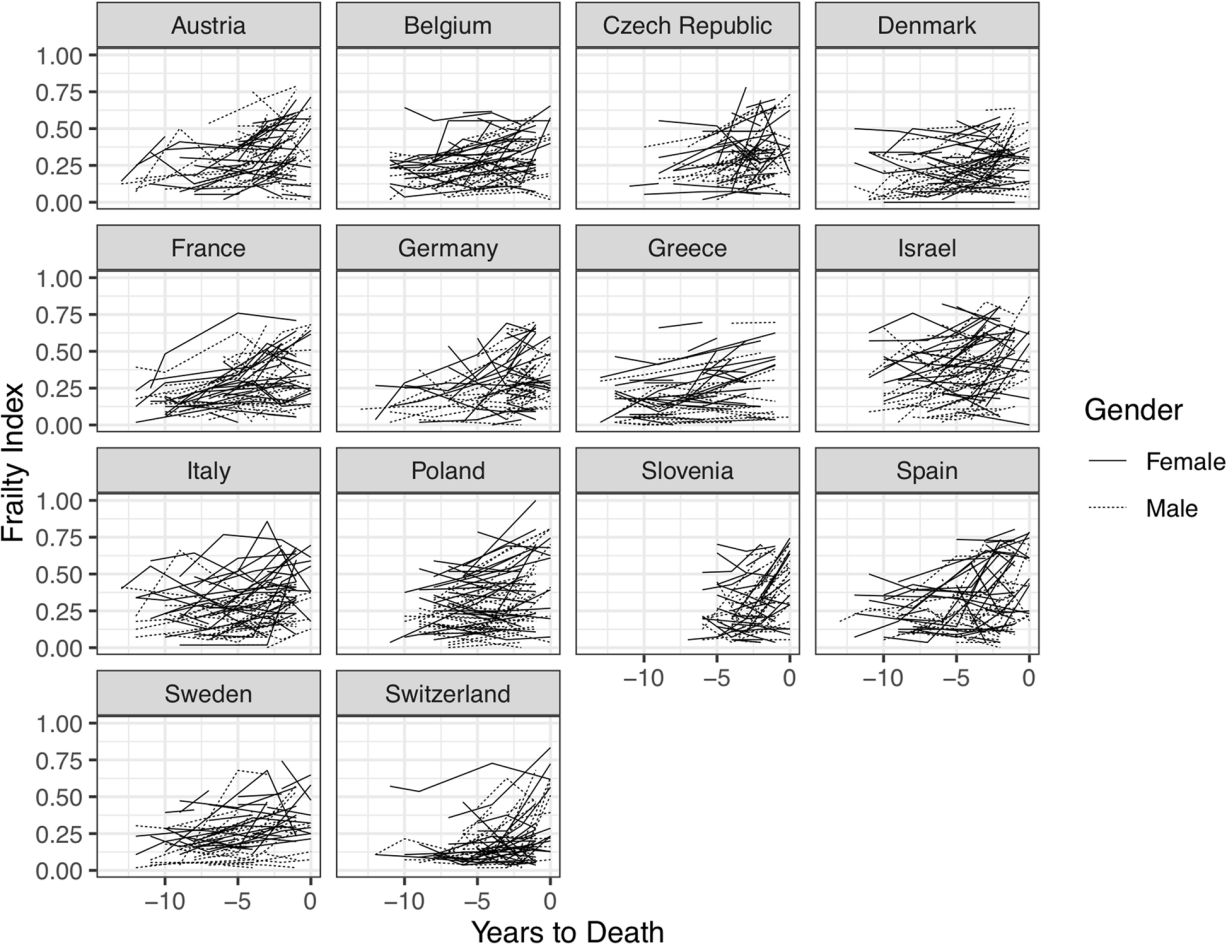
Spaghetti plots of FI trajectories in a random subset of 50 participants in each of the 14 countries

## Results

### Descriptive characteristics

Table 1 shows the sample characteristics in all countries. There were country differences in years of education, distribution of dementia incidence ${X}_{\left(14\right)}^{2}$=140.58, *p* < .001), and distribution of men and women (${X}_{\left(14\right)}^{2}$=$38.43$, *p* <.001). At study entry, FI levels also differed across countries (F_(14)=_2.10, *p* < .001), with Switzerland having the lowest mean FI (0.17 (SD=0.13)) and Israel, the highest (0.32 (SD = 0.20)). Individual observed trajectories of FI are shown in a randomly selected subset of participants for each country in Fig. 2.

### Differences across countries in frailty trajectories

 In all 14 countries, models estimating linear progression of frailty fitted the data best compared to models that estimated curvilinear trajectories according to comparison of BIC[30]. See Table 2 for BIC values for linear and quadratic models. These analyses suggest that, on average, frailty progresses at constant rate towards the end of life. Results of the linear models are presented in Table 3, and quadratic models in Table S2.

**Table 2:** BIC indices for quadratic and linear models

	BIC Indices at 6 months before death	
	Quadratic Model	Linear Model
**Northern**		
Denmark	5438.901	4607.310
Sweden	6193.397	5170.451
**Western**		
Austria	4722.989	4165.836
Belgium	6101.545	5397.893
France	5114.072	4489.354
Germany	2538.958	2296.394
Switzerland	2315.370	2011.174
**Eastern**		
Czech	5264.216	4792.537
Poland	3008.825	2571.088
**Southern**		
Italy	6985.237	5993.559
Spain	11228.581	10186.947
Greece	5503.197	4223.329
Slovenia	1821.028	1762.04
Israel	4508.526	4013.185

**Table 3:** Results from the final growth curve models fitted to frailty index measures in 14 countries participating in the SHARE study. Individual effects of baseline age, baseline age^2^, time to death, dementia, education, and gender are displayed for each of the fixed effects (Intercept (FI 6 months prior to death), and Linear growth rate (Yearly rate of change in FI)) in turn

	Northern		Western					Eastern		Southern				
	Sweden	Denmark	Austria	Belgium	France	Germany	Switzerland	Czech Republic	Poland	Italy	Spain	Greece	Slovenia	Israel
	*β*	*β*	*β*	*β*	*β*	*β*	*β*	*β*	*β*	*β*	*β*	*β*	*β*	*β*
	*(SE)*	*(SE)*	*(SE)*	*(SE)*	*(SE)*	*(SE)*	*(SE)*	*(SE)*	*(SE)*	*(SE)*	*(SE)*	*(SE)*	*(SE)*	*(SE)*
Fixed Effects:														
**Intercept (FI 6 months prior to death)**	0.229*** (0.014)	0.246*** (0.016)	0.252*** (0.015)	0.279*** (0.014)	0.267*** (0.012)	0.314*** (0.025)	0.213*** (0.015)	0.327*** (0.020)	0.311*** (0.019)	0.249*** (0.012)	0.251*** (0.010)	0.191*** (0.016)	0.245*** (0.027)	0.336*** (0.017)
Baseline Age (+1 year)	0.004** (0.001)	0.004*** (0.001)	0.005*** (0.001)	0.004*** (0.001)	0.004*** (0.001)	0.004** (0.001)	0.000 (0.001)	0.003* (0.001)	0.006*** (0.001)	0.007*** (0.001)	0.005*** (0.001)	0.010*** (0.001)	0.005* (0.002)	0.009*** (0.002)
Baseline Age^2^	0.000 (0.000)	0.000 (0.000)	0.000 (0.000)	0.000 (0.000)	0.000 (0.000)	0.000 (0.000)	0.000 (0.000)	0.000 (0.000)	0.000 (0.000)	0.000 (0.000)	0.000 (0.000)	0.000*** (0.000)	0.000 (0.000)	0.000 (0.000)
Time to Death (+1 year closer)	0.000 (0.002)	-0.002 0.002)	-0.006* (0.002)	-0.001 (0.002)	-0.003 (0.002)	-0.004 (0.003)	-0.003 (0.003)	-0.006* (0.003)	-0.001 (0.004)	-0.002 (0.002)	-0.004* (0.002)	0.001 (0.002)	0.007 (0.006)	-0.003 (0.003)
Dementia	0.197*** (0.026)	0.141*** (0.025)	0.196*** (0.026)	0.143*** (0.022)	0.134*** (0.024)	0.196*** (0.036)	0.241*** (0.038)	0.169*** (0.024)	0.176*** (0.035)	0.182*** (0.018)	0.165*** (0.014)	0.169*** (0.034)	0.175*** (0.031)	0.152*** (0.025)
Education (+1 year)	-0.005* (0.002)	-0.005* (0.002)	-0.006** (0.002)	-0.005* (0.002)	-0.006*** (0.002)	-0.009* (0.003)	-0.006** (0.002)	-0.014*** (0.002)	-0.010* (0.003)	-0.008*** (0.002)	-0.007*** (0.001)	-0.002 (0.002)	-0.006* (0.003)	-0.006* (0.002)
Female	0.026 (0.014)	-0.005 (0.013)	0.027 (0.016)	0.052*** (0.014)	0.033* (0.015)	0.021 (0.020)	-0.017 (0.018)	0.057*** (0.014)	0.054* (0.021)	0.086*** (0.014)	0.054*** (0.012)	0.057** (0.017)	0.028 (0.021)	0.018 (0.020)
**Linear growth rate (Yearly rate of change in FI)**	0.017*** (0.002)	0.013*** (0.003)	0.020*** (0.003)	0.021*** (0.003)	0.019*** (0.003)	0.031*** (0.005)	0.020*** (0.003)	0.020*** (0.003)	0.013* (0.005)	0.020*** (0.002)	0.017*** (0.002)	0.012*** (0.003)	0.006 (0.007)	0.017*** (0.004)
Baseline Age (+1 year)	0.001* (0.000)	0.000* (0.000)	0.000* (0.000)	0.001*** (0.000)	0.000* (0.000)	0.001* (0.000)	0.000 (0.000)	0.000 (0.000)	0.000 (0.000)	0.000* (0.000)	0.000* (0.000)	0.001*** (0.000)	0.001 (0.001)	0.000 (0.000)
Baseline Age^2^	0.000 (0.000)	0.000 (0.000)	0.000 (0.000)	0.000 (0.000)	0.000 (0.000)	0.000 (0.000)	0.000 (0.000)	0.000 (0.000)	0.000 (0.000)	0.000 (0.000)	0.000 (0.000)	0.000*** (0.000)	0.000 (0.000)	0.000 (0.000)
Time to Death (+1 year closer)	0.001** (0.000)	0.000 (0.000)	0.001* (0.000)	0.001*** (0.000)	0.001* (0.000)	0.001 (0.001)	0.001 (0.001)	0.001 (0.000)	0.000 (0.001)	0.002*** (0.000)	0.000 (0.000)	0.001 (0.000)	0.006*** (0.001)	0.001* (0.001)
Dementia	0.021*** (0.003)	0.020*** (0.003)	0.017*** (0.004)	0.015** (0.004)	0.012* (0.004)	0.018*** (0.005)	0.031*** (0.006)	0.022*** (0.005)	0.024*** (0.006)	0.017*** (0.003)	0.013*** (0.002)	0.014*** (0.004)	0.028* (0.012)	0.025*** (0.004)
Education (+1 year)	0.000 (0.000)	0.000 (0.000)	0.000 (0.000)	0.000 (0.000)	0.000 (0.000)	-0.001 (0.001)	-0.001 (0.000)	-0.001 (0.000)	0.000 (0.001)	0.000 (0.000)	0.000 (0.000)	0.001* (0.000)	0.000 (0.001)	0.000 (0.000)
Female	-0.002 (0.002)	-0.003 (0.002)	-0.003 (0.003)	-0.003 (0.002)	-0.003 (0.002)	-0.003 (0.003)	-0.008* (0.003)	0.004 (0.003)	-0.004 (0.003)	0.003 (0.002)	-0.004* (0.002)	0.000 (0.002)	-0.003 (0.007)	-0.003 (0.003)
Random Effects (Variances):														
**Intercept (FI 6 months prior to death)**	0.024*** (0.002)	0.018*** (0.002)	0.018*** (0.002)	0.023*** (0.002)	0.018*** (0.002)	0.022*** (0.003)	0.012*** (0.002)	0.019*** (0.002)	0.019*** (0.003)	0.019*** (0.002)	0.019*** (0.002)	0.024*** (0.003)	0.019*** (0.003)	0.027*** (0.003)
**Linear growth rate (Yearly rate of change in FI)**	0.000*** (0.000)	0.000** (0.000)	0.000* (0.000)	0.000*** (0.000)	0.000*** (0.000)	0.000* (0.000)	0.000 (0.000)	0.000* (0.000)	0.000 (0.000)	0.000* (0.000)	0.000 (0.000)	0.000*** (0.000)	0.000 (0.000)	0.000* (0.000)
Residual	0.006*** (0.001)	0.006*** (0.001)	0.011*** (0.001)	0.006*** (0.001)	0.008*** (0.001)	0.009*** (0.001)	0.007*** (0.001)	0.010*** (0.001)	0.013*** (0.002)	0.011*** (0.001)	0.016*** (0.001)	0.005*** (0.001)	0.009*** (0.002)	0.013*** (0.001)
Goodness of Fit (BIC):	5170.451	4607.31	4286.361	5397.893	4489.354	2296.394	2011.174	4792.537	2571.088	5993.559	10186.947	4223.329	1762.040	4013.185

*β *Coefficient, *FI *Frailty Index, *SE *Standard error

*p < .05; **p = .001; ***p < .0001

Israel was found to be the country with the highest level of frailty before death. On average, 6 months before death, the FI for a reference individual in Israel was estimated at 0.336 (SE = 0.017), with an annual rate of increase of 0.017 (SE = 0.004). In contrast, Greece was the country with the lowest frailty before death with the average FI for a reference individual estimated at 0.191 (SE = 0.016) with an annual rate of increase of 0.012 (SE = 0.003).

Frailty in Germany participants progressed at the fastest annual rate (0.031 (SE = 0.005)) whereas in Slovenia, rate of change was the slowest (0.006 (SE = 0.007)), although this estimate did not reach conventional statistical significance thresholds.

### Factors associated with FI and rate of change

**Fig. 3:**
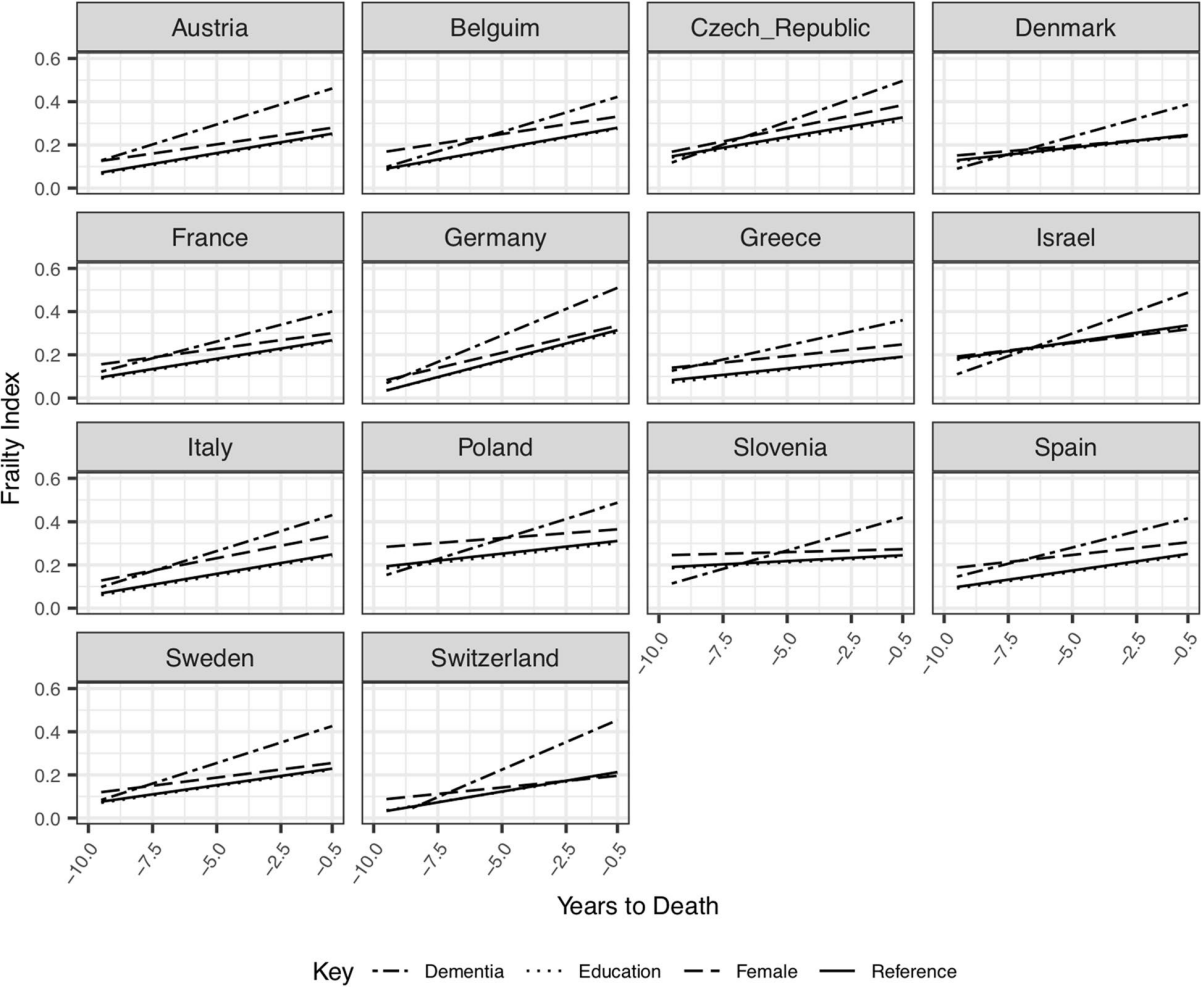
Model trajectories of FI and the impact of factors associated with rate of change in FI in the years prior to death

In all countries except Denmark and Switzerland, women were found to have higher levels of frailty near the time of death than men although estimates reached conventional statistical significance threshold levels only in half of the countries studied. In most countries, the direction of the association between sex/gender and rate of frailty progression suggests that frailty in women increased at a slower rate than in men, with estimates of the association reaching conventional statistical thresholds in Switzerland (0.008 (SE = 0.003)) and Spain (-0.004 (SE = 0.002)). Figure 3 compares model trajectories of FI and the impact of factors associated with rate of change in FI in the years prior to death across all 14 countries.

Higher education was associated with significantly lower levels of the FI in all countries, except Greece. Yet, Greece was the only country where education was associated with rate of change in frailty, with results indicating that per additional year of education the rate of annual increase in FI changed by *β *= 0.001 (SE = 0.000).

In addition, higher levels of frailty 6 months before death were observed in individuals diagnosed with dementia during follow up in all countries. This ranged from an increase in the FI of 0.134 (SE = 0.024) in France, to 0.241 (SE = 0.038 in Switzerland). A significantly faster rate of frailty progression prior to death was also observed in all countries in individuals who received a diagnosis of dementia. The smallest annual increase in rate of change in frailty prior to death in individuals who received a diagnosis of dementia occurred in France (*β* = 0.012 (SE = 0.004)), and the largest in Switzerland (*β* = 0.031 (SE = 0.006)).

Finally, older age at study entry was found to be associated with significantly higher levels of frailty before death in all countries except for Switzerland. The association of baseline age with rate of change in frailty was found to be significant in most of the countries but not all. Our results did not identify a non-linear effect of age at study entry on levels or rate of change of FI.

## Discussion

Our study showed an increase in frailty towards the end of life although we found no evidence of acceleration in frailty rate as individuals approach death. Dementia diagnosis, and baseline age were both associated with a faster increase in FI in the years prior to death. Women, were found to have a higher level of frailty but a slower decline (indicated by a slower increase in FI) in the years prior to death than men, although this was only significant in two of the fourteen countries.

Evidence of frailty progression in the years prior to death is scarce [14]. A recent study by Stow et al. examined trajectories of frailty in the year preceding death, identifying three trajectories of deficit accumulation: rapidly increasing, moderately increasing, and stable. Other studies examining end of life trajectories in frail individuals suggest that decline is a slow and gradual process occurring over the period of many years [15]. Our independent analysis of data from 14 European countries adds to this literature generating evidence about various aspects of frailty changes and the factors that may influence progression in the years preceding death. However, the low number of individuals with more than three time points potentially hampers our results. It may be the case that a greater number of individuals with three or more time points is required to provide definitive evidence of a non-linear trajectory of frailty.

### Trajectory shapes and geographical differences

Our results indicate that rate of frailty change does not accelerate as individuals approach death. Indeed, in 13 of 14 countries positive linear frailty progression was estimated as individuals approach death, whilst a positive linear progression of frailty was estimated in Slovenia it did not reach significance. Recent studies have estimated frailty trajectories before death, though because of differences in methodologies used, the comparison of their results with ours is not straightforward. For example, Ward et al. used k-means to identify subgroups of US veterans whose frailty evolution follows different non-linear patterns of change [33]. They used splines to estimate the non-linear trajectories but did not contrast model fit with models that allowed them to test whether frailty progressed linearly or not. Stolz et al., instead, used data from the HRS study to estimate non-linear frailty trajectories before death using a change point model [16]. They showed that the normal (pre-terminal) rate of health deficit accumulation increased five-fold (terminal decline) about three years before death. Terminal decline was steeper and occurred later in men compared to women. Importantly, the follow-up period in the HRS cohort is longer than the current study, with time to death ranging from 0 to 21 years. It is possible that a longer follow-up period would have allowed us to capture acceleration in SHARE.

We identified differences in level and rate of change in frailty across countries, although not consistent with previous reports that Southern European countries had increased levels of frailty [3]. For instance, in our analyses, Israel was the country with the highest mean level of frailty before death whilst Germany was the country with the fastest increase in frailty. It is possible that the lower years of education in Israel relative to the average years of education in the rest of the analytical sample, explains these differences. Germany was the country with the fastest rate of frailty increase despite no obvious differences in the covariates considered in our analyses with the other countries. These findings suggest that, as reported by Stolz et al. (2017) [3], wealth and income may not be the strongest determinants of frailty progression at the end of life. However, the direct interpretation of these differences requires caution, as important conceptual and methodological differences limit the direct comparison of findings previously reported in SHARE [3, 13, 34–36]. Previous published studies using SHARE data analysed ageing-related changes, instead of changes in the years prior to death, and hence differences are expected. Other differences between published reports and ours include the samples selected for analyses (differences in age and treatment of dementia cases), adjustment for different sets of covariates, years of follow up and number of time points. Yet, despite these expected differences in reports, our results are in partial agreement with previous SHARE research on FI. For instance, in relation to the shape of the estimated curves, Walkden, Anderson [1] reported linear but not quadratic increases in frailty over nine years in migrant and non-migrant groups [34], whereas Stolz, Mayerl [2] identified a quadratic curve in an investigation of the impact of occupational class and wealth on frailty changes [3].

### Factors associated with FI level and rate of change

Our results regarding sex/gender differences in FI progression as individuals approach death are largely consistent with previous findings that women have higher levels of FI before death consistent with the ‘male-female health survival paradox’ [8, 37]. These findings agree with the results reported in a meta-analysis [9] that reported that females had higher FI scores than males, tolerating frailty better. Interestingly, whilst FI remained consistently higher in women than men, our results show that women accumulated deficits slower than men in the years preceding death, it is possible that this contributes to the ability of women to tolerate frailty better. Numerous factors have been postulated as possible explanations of sex/gender differences in frailty progression, including biological and psychosocial factors and differences in self reporting of health [38, 39]. An in-depth discussion of these factors is beyond the scope of this paper, but an informative exposition can be found in Gordon et al. [9].

Our results about the effect of education on frailty levels were consistent across all countries included in our study, showing that more educated individuals had fewer deficits before death than individuals with less education. These findings are in agreement with results reported by Stolz et al. (2017) [3]. Jointly, these findings demonstrate the long-term protective effect of education in relation to the accumulation of deficits that may be the result of a life course engagement in healthier behaviours, improved access to health care and better use of available resources to preserve health [40–42].

The results of our study have practical implications. Frailty can be targeted by interventions up to very old age. Understanding how frailty develops at the end of life may help to identify the stage of frailty that is irreversible, and where palliative care would be more appropriate [18]. Moreover, interventions can be better targeted if we gain insight into factors that are associated with certain frailty trajectories at the end of life.

The analyses also indicated that individuals who developed dementia were found to have higher frailty and steeper trajectories of frailty before death when compared with individuals who remained dementia free across the study. These findings correspond with a growing body of literature linking frailty with dementia [43], and further highlight that frailty should be considered in the clinical care and management of these neurological conditions [44]. Further understanding of frailty trajectories may also help in predicting and preventing dementia across our older populations [45].

### Strengths and limitations

Our analyses have various strengths and limitations. It is the first study to consistently look at terminal changes in frailty in 14 harmonised samples. The coordinated analytical approach employed here allowed us to perform a fair comparison of results across countries, eliminating potential biases due to differences in the derivation of the FI and study design. Despite the relatively short time elapsed between data collection waves, it is possible that opportunities to detect acceleration in terminal changes were hampered. Firstly, end of life questionnaires are completed by a proxy respondent which may be less reliable than health records. It is also possible that differences across countries in the recording of year of death exist that could also introduce bias in our results. Furthermore, it was not possible to examine cause of death, it is possible that these may result in distinct trajectories, some of which may reveal acceleration in terminal changes. Future research is required that explores the cause of death in more detail and focusses on the influence of diseases that can progress to a terminal state such as kidney disease, heart failure, or AIDS. However, our use of harmonised end of life interviews to derive data about age of death increases our confidence in results. Differences across countries in policies and practices of institutionalisation of older adults, who are excluded from the study, may also result in an underestimation of frailty changes in the general population. Finally, data collection regarding sex/gender is unclear making the distinction between biological sex and gender unfeasible. This necessitated our approach to combine the associations of sex and gender. In the context of frailty both biological sex and gender are relevant, and may influence prevalence and disease progression; however, they are not interchangeable. This important issue should be addressed in subsequent waves.

In conclusion, we observed an increase in frailty towards the end of life among older Europeans, but we did not find evidence for acceleration in its rate of progression as individuals approach death. Furthermore, we identified factors that are associated with (a) higher levels of frailty prior to death, including female sex/gender, dementia, and lower level of education, and (b) frailty progression in the years prior to death, including dementia and age. Knowledge of these influencing factors may support the development of personalized care pathways at the end of life.

## Supplementary Information

**Additional file 1.**

## Data Availability

The datasets analysed during the current study are available in the SHARE, details of which can be found elsewhere [22, 23]. R and MPlus scripts for the analysis can be made available upon request to the corresponding author (MW).

## Abbreviations

BIC: Bayesian Information Criterion
ELSE: English Longitudinal Study of Ageing
FI: Frailty index
HRS: Health and Retirement Study
SHARE: Survey of Health, Aging and Retirement Study in Europe
