# Supplementary material for: Frailty trajectories and associated factors in the years prior to death: evidence from 14 countries in the Survey of Health, Aging and Retirement in Europe
Source: BMC Geriatr. 2023 Jan 27;23:49. doi: 10.1186/s12877-023-03736-1 (PMC9881297; doi:10.1186/s12877-023-03736-1)
Supplement: Supplementary file 1 — Additional file 1. [file 12877_2023_3736_MOESM1_ESM.docx]

**Supplementary Material: Trajectories of in Frailty in the years prior to death: evidence from 14 countries in the Survey of Health, Aging and Retirement in Europe**

**Table S1: List of 56 Variables included in the Frailty Index** 2

**Table S2: Results from the quadratic growth curve models fitted to frailty index measures in 14 countries participating in the SHARE study** 3

**Table S1: List of 56 Variables included in the Frailty Index**

| **List of 56 Variables included in the frailty index** | **Cut Point** |
| --- | --- |
|  |  |
| Hospitalisation in Past Year | Yes = 1, No =0 |
| Self-Rated Health | Fair, Poor = 1, Excellent, Very good, Good = 0 |
| Comorbidities: |  |
| Heart Attack | Yes = 1, No =0 |
| Chronic Lung Disease | Yes = 1, No =0 |
| Parkinson Disease | Yes = 1, No =0 |
| Stroke or CVA | Yes = 1, No =0 |
| Cancer | Yes = 1, No =0 |
| Cataracts | Yes = 1, No =0 |
| High Blood Cholesterol | Yes = 1, No =0 |
| Long-term illness | Yes = 1, No =0 |
| High blood pressure | Yes = 1, No =0 |
| Diabetes mellitus or high blood sugar | Yes = 1, No =0 |
| Arthritis | Yes = 1, No =0 |
| Stomach or duodenal ulcer | Yes = 1, No =0 |
| Hip or femoral fracture | Yes = 1, No =0 |
| Signs and Symptoms: |  |
| Falling down | Yes = 1, No =0 |
| Dizziness | Yes = 1, No =0 |
| Trouble sleeping | Yes = 1, No =0 |
| Hearing | Fair, Poor, Deaf = 1, Excellent, Very good, Good = 0 |
| Pain in any joint | Yes = 1, No =0 |
| Eyesight distance | Fair, Poor = 1, Excellent, Very good, Good = 0 |
| Eyesight reading | Fair, Poor = 1, Excellent, Very good, Good = 0 |
| Function: |  |
| Climbing several flights of stairs | Yes = 1, No =0 |
| Pushing or pulling large objects | Yes = 1, No =0 |
| Dressing | Yes = 1, No =0 |
| Eating | Yes = 1, No =0 |
| Using a map to get around | Yes = 1, No =0 |
| Stooping/kneeling/crouching | Yes = 1, No =0 |
| Managing money | Yes = 1, No =0 |
| Lifting/carrying weights over 5kg | Yes = 1, No =0 |
| Getting in or out of bed | Yes = 1, No =0 |
| Walking across a room | Yes = 1, No =0 |
| Making telephone calls | Yes = 1, No =0 |
| Sitting for about 2 hours | Yes = 1, No =0 |
| Walking 100m | Yes = 1, No =0 |
| Getting up from a chair | Yes = 1, No =0 |
| Preparing a hot meal | Yes = 1, No =0 |
| Taking medications | Yes = 1, No =0 |
| Bathing or showering | Yes = 1, No =0 |
| Reaching or extending arms | Yes = 1, No =0 |
| Picking up a coin from a small table | Yes = 1, No =0 |
| Using the toilet | Yes = 1, No =0 |
| Shopping for groceries | Yes = 1, No =0 |
| Doing housework/gardening | Yes = 1, No =0 |
| Limitations with activities | Severely limited, Limited but not severely = 1, Not limited = 0 |
| Frequency of vigorous activity | One to three times a month, Hardly ever/never = 1, More than once a week, Once a week = 0 |
| Frequency of moderate activity | One to three times a month, Hardly ever/never = 1, More than once a week, Once a week = 0 |
| Mental well-being: |  |
| Appetite | Yes = 1, No =0 |
| Suicidality | Yes = 1, No =0 |
| Lack of enjoyment | Yes = 1, No =0 |
| Fatigue | Yes = 1, No =0 |
| Fear of falling down | Yes = 1, No =0 |
| Depression | Yes = 1, No =0 |
| Pessimism | Yes = 1, No =0 |
| Loss of Interest | Yes = 1, No =0 |
| Concentration | Yes = 1, No =0 |

Note. CVA, Cardiovascular accident

**Table S2: Results from the quadratic growth curve models fitted to frailty index measures in 14 countries participating in the SHARE study**

|  |  | Northern | |  | | Western | | | | |  | | Eastern | |  | | Southern | | | |  | |  |
| --- | --- | --- | --- | --- | --- | --- | --- | --- | --- | --- | --- | --- | --- | --- | --- | --- | --- | --- | --- | --- | --- | --- | --- |
|  | Sweden | | Denmark | | Austria | | Belgium | France | Germany | Switzerland | | Czech Republic | | Poland | | Italy | | Spain | Greece | Slovenia | | Israel | |
|  | $\beta$ | | $\beta$ | | $\beta$ | | $\beta$ | $\beta$ | $\beta$ | $\beta$ | | $\beta$ | | $\beta$ | | $\beta$ | | $\beta$ | $\beta$ | $\beta$ | | $\beta$ | |
|  | $(SE)$ | | $(SE)$ | | $(SE)$ | | $(SE)$ | $(SE)$ | $(SE)$ | $(SE)$ | | $(SE)$ | | $(SE)$ | | $(SE)$ | | $(SE)$ | $(SE)$ | $(SE)$ | | $(SE)$ | |
| Fixed Effects: |  | |  | |  | |  |  |  |  | |  | |  | |  | |  |  |  | |  | |
| Intercept | 0.231*** (0.016) | | 0.241*** (0.018) | | 0.258*** (0.016) | | 0.288*** (0.015) | 0.261*** (0.013) | 0.322*** (0.029) | 0.220*** (0.017) | | 0.326*** (0.018) | | 0.332*** (0.027) | | 0.261*** (0.013) | | 0.264*** (0.011) | 0.234*** (0.022) | 0.257*** (0.029) | | 0.331*** (0.021) | |
| Baseline Age  (+1 year) | 0.004** (0.001) | | 0.005*** (0.001) | | 0.006*** (0.001) | | 0.005*** (0.001) | 0.005*** (0.001) | 0.004* (0.001) | 0.001 (0.002) | | 0.003* (0.001) | | 0.005* (0.002) | | 0.006*** (0.001) | | 0.006*** (0.001) | 0.011*** (0.002) | 0.004* (0.002) | | 0.010*** (0.002) | |
| Baseline Age^2^ | 0.000 (0.000) | | 0.000 (0.000) | | 0.000 (0.000) | | 0.000 (0.000) | 0.000 (0.000) | 0.000 (0.000) | 0.000 (0.000) | | 0.000 (0.000) | | 0.000 (0.000) | | 0.000 (0.000) | | 0.000 (0.000) | 0.000** (0.000) | 0.000 (0.000) | | 0.000 (0.000) | |
| Time to Death  (+1 year closer) | 0.001 (0.002) | | -0.003 0.002) | | -0.008* (0.003) | | -0.003 (0.002) | -0.004 (0.002) | -0.003 (0.003) | -0.007 (0.004) | | -0.007* (0.003) | | -0.003 (0.005) | | -0.003 (0.002) | | -0.007*** (0.002) | 0.003 (0.003) | 0.003 (0.007) | | 0.000 (0.003) | |
| Dementia | 0.229*** (0.029) | | 0.171*** (0.028) | | 0.206*** (0.027) | | 0.179*** (0.021) | 0.183*** (0.027) | 0.227*** (0.037) | 0.275*** (0.041) | | 0.174*** (0.025) | | 0.171*** (0.040) | | 0.176*** (0.022) | | 0.170*** (0.016) | 0.144*** (0.034) | 0.190*** (0.033) | | 0.158*** (0.031) | |
| Education  (+1 year) | -0.005* (0.002) | | -0.005* (0.002) | | -0.006* (0.002) | | -0.005* (0.002) | -0.005* (0.002) | -0.0100* (0.004) | -0.007*** (0.002) | | -0.012*** (0.002) | | -0.011* (0.004) | | -0.008*** (0.002) | | -0.008*** (0.001) | -0.001 (0.003) | -0.007* (0.003) | | -0.007* (0.003) | |
| Female | 0.024 (0.016) | | 0.002 (0.015) | | 0.030 (0.018) | | 0.038* (0.015) | 0.024 (0.016) | 0.019 (0.022) | -0.021 (0.020) | | 0.058*** (0.016) | | 0.037 (0.028) | | 0.091*** (0.016) | | 0.046*** (0.013) | 0.066* (0.023) | 0.033 (0.022) | | 0.020 (0.024) | |
| Linear growth rate | 0.020* (0.007) | | 0.020* (0.009) | | 0.030** (0.009) | | 0.036*** (0.008) | 0.020* (0.008) | 0.053* (0.019) | 0.030* (0.010) | | 0.025* (0.011) | | 0.038* (0.017) | | 0.039*** (0.008) | | 0.034*** (0.007) | 0.053*** (0.012) | 0.033 (0.019) | | 0.017 (0.012) | |
| Baseline Age  (+1 year) | 0.001 (0.001) | | 0.001* (0.000) | | 0.002* (0.001) | | 0.001 (0.000) | 0.000 (0.000) | 0.000 (0.001) | 0.000 (0.001) | | 0.000 (0.001) | | 0.000 (0.001) | | 0.000 (0.001) | | 0.001 (0.001) | 0.001* (0.001) | 0.000 (0.002) | | 0.001 (0.001) | |
| Baseline Age^2^ | 0.000 (0.000) | | 0.000 (0.000) | | 0.000 (0.000) | | 0.000 (0.000) | 0.000 (0.000) | 0.000 (0.000) | 0.000 (0.000) | | 0.000 (0.000) | | 0.000 (0.000) | | 0.000 (0.000) | | 0.000 (0.000) | 0.000 (0.000) | 0.000 (0.000) | | 0.000 (0.000) | |
| Time to Death  (+1 year closer) | 0.001 (0.001) | | 0.001 (0.001) | | 0.000 (0.001) | | 0.000 (0.001) | 0.001 (0.001) | 0.003 (0.002) | 0.000 (0.002) | | 0.000 (0.002) | | -0.001 (0.003) | | 0.003* (0.001) | | 0.000 (0.001) | 0.004* (0.001) | 0.004 (0.004) | | 0.003 (0.002) | |
| Dementia | 0.040* (0.013) | | 0.050*** (0.012) | | 0.019 (0.011) | | 0.050*** (0.010) | 0.058*** (0.013) | 0.049 (0.025) | 0.060* (0.020) | | 0.019 (0.015) | | 0.016 (0.028) | | 0.01 (0.014) | | 0.014 (0.009) | 0.002 (0.016) | 0.108*** (0.022) | | 0.016 (0.016) | |
| Education  (+1 year) | 0.000 (0.001) | | 0.000 (0.001) | | 0.000 (0.001) | | -0.001 (0.001) | 0.001 (0.001) | -0.003 (0.002) | -0.001 (0.001) | | 0.001 (0.001) | | -0.002 (0.003) | | 0.000 (0.001) | | -0.001 (0.001) | 0.001 (0.001) | -0.004 (0.002) | | 0.000 (0.001) | |
| Female | -0.005 (0.006) | | -0.004 (0.006) | | 0.002 (0.010) | | -0.023** (0.007) | -0.017* (0.008) | -0.004 (0.013) | -0.009 (0.011) | | 0.009 (0.009) | | -0.018 (0.015) | | 0.008 (0.008) | | -0.011 (0.007) | -0.002 (0.008) | 0.005 (0.015) | | -0.004 (0.011) | |

Note. $\beta$, Coefficient; SE, Standard error

**p* < .05; ***p* = .001; ****p* < .0001

***Table S2 Continued***

|  |  | Northern | |  | | Western | | | | |  | | Eastern | |  | | Southern | | | |  | |  |
| --- | --- | --- | --- | --- | --- | --- | --- | --- | --- | --- | --- | --- | --- | --- | --- | --- | --- | --- | --- | --- | --- | --- | --- |
|  | Sweden | | Denmark | | Austria | | Belgium | France | Germany | Switzerland | | Czech Republic | | Poland | | Italy | | Spain | Greece | Slovenia | | Israel | |
|  | $\beta$ | | $\beta$ | | $\beta$ | | $\beta$ | $\beta$ | $\beta$ | $\beta$ | | $\beta$ | | $\beta$ | | $\beta$ | | $\beta$ | $\beta$ | $\beta$ | | $\beta$ | |
|  | $(SE)$ | | $(SE)$ | | $(SE)$ | | $(SE)$ | $(SE)$ | $(SE)$ | $(SE)$ | | $(SE)$ | | $(SE)$ | | $(SE)$ | | $(SE)$ | $(SE)$ | $(SE)$ | | $(SE)$ | |
| Fixed Effects: |  | |  | |  | |  |  |  |  | |  | |  | |  | |  |  |  | |  | |
| Quadratic growth rate | 0.001  (0.001) | | 0.003*  (0.001) | | 0.002  (0.002) | | 0.003*  (0.001) | 0.001  (0.001) | 0.006  (0.003) | 0.003  (0.002) | | 0.001  (0.002) | | 0.004  (0.003) | | 0.004*  (0.001) | | 0.003*  (0.001) | 0.005**  (0.002) | 0.008  (0.004) | | 0.000  (0.002) | |
| Baseline Age  (+1 year) | 0.000  (0.000) | | 0.000  (0.000) | | 0.000*  (0.000) | | 0.000  (0.000) | 0.000  (0.000) | 0.000  (0.000) | 0.000  (0.000) | | 0.000  (0.000) | | 0.000  (0.000) | | 0.000  (0.000) | | 0.000  (0.000) | 0.000  (0.000) | 0.000  (0.000) | | 0.000  (0.000) | |
| Baseline Age^2^ | 0.000  (0.000) | | 0.000  (0.000) | | 0.000  (0.000) | | 0.000  (0.000) | 0.000  (0.000) | 0.000  (0.000) | 0.000  (0.000) | | 0.000  (0.000) | | 0.000  (0.000) | | 0.000  (0.000) | | 0.000  (0.000) | 0.000  (0.000) | 0.000  (0.000) | | 0.000  (0.000) | |
| Time to Death  (+1 year closer) | 0.000  (0.000) | | 0.000*  (0.000) | | 0.000  (0.000) | | 0.000  (0.000) | 0.000  (0.000) | 0.001  (0.000) | 0.001  (0.000) | | 0.000  (0.000) | | 0.000  (0.000) | | 0.001**  (0.001) | | 0.000  (0.000) | 0.000*  (0.000) | 0.001  (0.001) | | 0.000  (0.000) | |
| Dementia | 0.002  (0.002) | | 0.002  (0.002) | | 0.000  (0.002) | | 0.004*  (0.002) | 0.005**  (0.002) | 0.003  (0.004) | 0.002  (0003) | | -0.002  (0.003) | | -0.001  (0.004) | | 0.000  (0.002) | | -0.001  (0.001) | -0.002  (0.002) | 0.023***  (0.006) | | -0.003  (0.002) | |
| Education  (+1 year) | 0.000  (0.000) | | 0.000  (0.000) | | 0.000  (0.000) | | 0.000  (0.000) | 0.000  (0.000) | 0.000  (0.000) | 0.000  (0.000) | | 0.000  (0.000) | | 0.000  (0.000) | | 0.000  (0.000) | | 0.000  (0.000) | 0.000  (0.000) | -0.001*  (0.001) | | 0.000  (0.000) | |
| Female | -0.001  (0.001) | | 0.000  (0.001) | | 0.001  (0.001) | | -0.003*  (0.001) | -0.002*  (0.001) | 0.000  (0.002) | 0.000  (0.002) | | 0.002  (0.002) | | -0.001  (0.002) | | 0.001  (0.001) | | -0.001  (0.001) | -0.001  (0.001) | 0.001  (0.003) | | 0.000  (0.001) | |
|  |  | |  | |  | |  |  |  |  | |  | |  | |  | |  |  |  | |  | |
| Random Effects: |  | |  | |  | |  |  |  |  | |  | |  | |  | |  |  |  | |  | |
| Intercept | 0.024  (0.003)*** | | 0.019***  (0.002) | | 0.022***  (0.003) | | 0.022***  (0.002) | 0.019***  (0.002) | 0.021***  (0.003) | 0.013***  (0.002) | | 0.022***  (0.003) | | 0.023***  (0.005) | | 0.020***  (0.002) | | 0.021***  (0.002) | 0.024***  (0.004) | 0.023***  (0.003) | | 0.028***  (0.006) | |
| Linear growth rate | 0.000  (0.000) | | 0.000  (0.000) | | 0.000  (0.000) | | 0.000  (0.000) | 0.000  (0.001) | 0.000  (0.001) | 0.000  (0.000) | | 0.000  (0.001) | | 0.000  (0.002) | | 0.000  (0.000) | | 0.000  (0.000) | 0.000  (0.001) | 0.000  (0.001) | | 0.000  (0.002) | |
| Quadratic growth rate | 0.000  (0.000) | | 0.000  (0.000) | | 0.000  (0.000) | | 0.000  (0.000) | 0.000  (0.000) | 0.000  (0.000) | 0.000  (0.000) | | 0.000  (0.000) | | 0.000  (0.000) | | 0.000  (0.000) | | 0.000  (0.000) | 0.000  (0.000) | 0.000  (0.000) | | 0.000  (0.000) | |
| Residual | 0.006***  (0.001) | | 0.005***  (0.001) | | 0.010***  (0.001) | | 0.006***  (0.000) | 0.007***  (0.001) | 0.008***  (0.002) | 0.006***  (0.001) | | 0.009***  (0.001) | | 0.012***  (0.002) | | 0.010***  (0.001) | | 0.014***  (0.001) | 0.004***  (0.001) | 0.007***  (0.002) | | 0.012***  (0.002) | |
| Goodness of Fit (BIC): | 6193.397 | | 5438.901 | | 4722.989 | | 6101.545 | 5114.072 | 2538.958 | 2315.370 | | 5264.216 | | 3008.825 | | 6985.237 | | 1128.581 | 5503.197 | 1821.028 | | 4508.526 | |

Note. $\beta$, Coefficient; SE, Standard error

**p* < .05; ***p* = .001; ****p* < .0001
